# A multi-center study of high dose Aldesleukin (Proleukin^® ^(HD IL-2) + Vemurafenib Zelboraf^® ^) therapy in patients with BRAF^V600 ^mutation positive metastatic melanoma (proclivity 01)

**DOI:** 10.1186/2051-1426-2-S3-P77

**Published:** 2014-11-06

**Authors:** Joseph Clark, Lawrence Flaherty, Marc Ernstoff, Henry Koon, Mohammed Milhem, Gerald Militello, Sanjiv Agarwala, Brendan Curti, Lee Cranmer, Christopher D Lao, Theodore F Logan, Jose Lutzky, Venkatesh Rudrapatna, Gregory Daniels, Bret Taback, Sandra Aung, James Lowder, David Lawson

**Affiliations:** 1 Loyola University, Maywood, IL, USA; 2 Karmanos Cancer Center, Detroit, MI, USA; 3 Dartmouth University, Hanover, NH, USA; 4 CWRU University Hospitals, Cleveland, OH, USA; 5 University of Iowa, Iowa City, IA, USA; 6 Hematology/Oncology Clinic, USA; 7 Saint Luke's Cancer Center, Center Valley, PA, USA; 8 Earle A. Chiles Research Institute, Portland, OR, USA; 9 University of Arizona Cancer Center, Tucson, AZ, USA; 10 University of Michigan, Ann Arbor, MI, USA; 11 Indiana University, Indianapolis, IN, USA; 12 University of Miami Medical Center, Miami, FL, USA; 13 University of Minnesota, Minneapolis, MN, USA; 14 Moores Cancer Center, La Jolla, CA, USA; 15 Columbia University, New York, NY, USA; 16 Prometheus Laboratories, San Diego, CA, USA; 17 Winship Cancer Institute, Emory University, Atlanta, GA, USA

## Purpose

To investigate whether the Vemurafenib-induced increased tumor antigen expression, T lymphocyte infiltration and tumor debulking improve the complete response rate induced by HD IL-2 in metastatic melanoma and if there is synergistic toxicity using the drugs in close approximation.

## Schema

**Figure 1:**
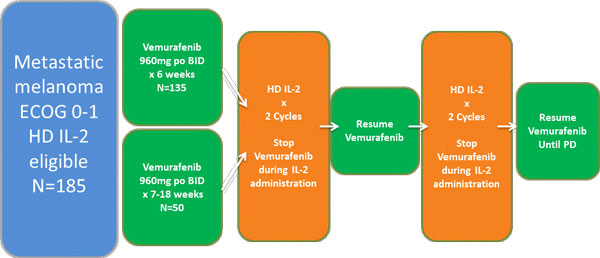
**Treatment of metastatic melanoma with HD IL-2 immunotherapy and targeted agent vemurafenib**.

Adult patients with measurable metastatic or unresectable Stage III melanoma with no prior therapy and a BRAF^V600 ^mutation who are candidates for HD IL-2 are eligible for entry into the first cohort of 135 patients (figure 1). Six weeks of Vemurafenib therapy per package insert precedes up to 2 courses of HD IL-2. Vemurafenib is administered during the outpatient intervals between cycles of HD IL-2 and following completion. A second cohort of up to 50 similar patients already responding or stable with < 18 weeks of Vemurafenib therapy will also be accrued. The study was amended to permit prior anti-PD-1 therapy. The primary endpoint is Complete Response (CR) and near CR at 6 months of therapy.

## Current status

Sixteen sites have enrolled patients. 41 patients have been enrolled to date, 27 in Cohort 1 and 14 in cohort 2. The Data Safety and Monitoring Board performed an initial safety analysis after the initial 8 patients which demonstrated no unexpected safety signal. An analysis of the effect of the combination on Progression Free Survival in both cohorts will be performed after the first 20% of patients in Cohort 1 have received at least one course of HD IL-2. The results of this analysis should be available at the time of the SITC meeting.

